# Computerized Neurocognitive Test (CNT) in mild cognitive impairment and Alzheimer's disease

**DOI:** 10.1590/S1980-57642014DN82000005

**Published:** 2014

**Authors:** Maira Okada de Oliveira, Sonia Maria Dozzi Brucki

**Affiliations:** Cognitive Neurology and Behavioral Group of the Department of Neurology of the University of São Paulo, SP, Brazil.

**Keywords:** computerized neurocognitive tests, computerized neuropsychological tests, Alzheimer's disease, mild cognitive impairment, elderly, cognition

## Abstract

Currently, computerized batteries are of great value in detecting cognitive impairment. This aim of this review was to compare the computerized neurocognitive batteries used in most studies with cognitive decline over the last 10 years. Using the search words computerized cognitive assessment with: dementia, mild cognitive impairment, and Alzheimer's disease, the CogState, CNS Vital Sings, COGDRAS and Mindstreams batteries were retrieved.

## Abstract (pt)

Atualmente, baterias de testes computadorizados têm sido de grande valor na detecção de comprometimento cognitivo. Esta revisão teve como objetivo comparar as baterias cognitivas computadorizadas que foram utilizadas nos últimos 10 anos, na maioria dos estudos com declínio cognitivo. Usando as palavras avaliação cognitiva computadorizada com: demência, comprometimento cognitivo leve e doença de Alzheimer nós encontramos as baterias CogState, CNS Vital Signs, COGDRAS e Mindstreams.

## INTRODUCTION

The use of Computerized neurocognitive tests (CNT) to evaluate cognition has been widely studied and may be the most suitable tool for the early detection of impairments.^1^ CNT are able to measure mild degrees of cognitive impairment and can gauge the effectiveness of an intervention.^2^ Computerized batteries offer a number of advantages over paper-and-pencil type tests: they are precise, accurate and can be timed to the nearest millisecond. In addition they are easy to administer and score, have greater standardization and multiple parallel versions may also be available, known to reduce practice effects.^3^

The advantages of a computerized battery are that it can be applied at bedside using a tablet device, results can be instant, and the tests applied by any person, eliminating examiner effects and providing increased reliability. The battery is also language independent and can be used in patients with mild aphasia offering consistent administration and scoring, while computerized tests can also generate alternative forms for repeated testing.^2,4,5^

The disadvantages include the absence of the active participation of consulting neuropsychologists analyzing the qualitative performance, and that the limitations in the ability to understand and manipulate information technology can cause a negative effect because elderly tend not to be familiar with it.^2,6^

The aim of this review was to compare the features of the computerized neuropsychological batteries used in most studies involving the cognitively impaired over the last 10 years, in order to verify which are most suitable for use in clinical practice within an outpatient clinic.

## METHODS

We examined on-line articles published between 2004 and 2014 on the PubMed database or the references of these articles, which cited mainly reliability and validity studies prior to 2004, searching for the most used and most cited battery for dementia, Alzheimer's disease (AD) and Mild Cognitive Impairment (MCI). The descriptors computerized cognitive assessment with: dementia, mild cognitive impairment and Alzheimer's disease were used. Only studies available in English were included.

**Exclusion criteria.** Articles published before 2004, those addressing other dementias or computer programs used for rehabilitation, were excluded as were articles unavailable online.

## RESULTS

A total of 101 articles published between 2004 and 2014 were identified. Sixty-one articles were excluded for not assessing patients with AD and MCI or because the computerized test was cited only once. Some articles appeared more than once. Articles were divided by tests, population, mean age, diagnosis and main results (Table 1) and organized according to the main characteristics of the tests (Table 2).

**Table 1:** Articles divided by tests, author, population, mean age, diagnosis and main results.

Tests	Authors	Population (n)	Age Mean (SD)	Diagnosis (n)	Results
CogState	Lim et al., 2013	HC: 105aMCI: 48AD: 42	HC: 73.62 (6.86)aMCI: 78.87 (6.92)AD: 79.57 (6.61)	HC aMCI AD	High test-retest reliability and stability in all groups and adequate to detect AD-related cognitive impairment
	Darby et al., 2012	263 older adults	64.6 (7)	HC	Repetitive assessment indicating a population with risk of decline
	Hammers et al., 2012	HC: 22MCI: 16AD: 37DLB: 5FTD: 7	70.5( 8.7)	HC, MCI, AD, DLB, FTD	Sensitivity, but not specificity, for diagnosing dementia
	Hammers et al., 2011	HC: 23 MCI: 20AD: 52DLB: 10FTD: 9	HC: 68.4 (9.5)MCI: 73.5 (5.9)AD: 70.8 (8.7)DLB: 70.4 (8.5)FTD: 64.2 (8.1)	HC, MCI, AD, DLB, FTD	Sensitive for cognitive impairment in established dementia and minimal practice effects at short test-retest intervals
CNS Vital Signs	Gualtieri and Johnson, 2006	1069	7-90	HC, neuropsychiatric patients, MCI and dementia	Psychometric characteristics and reliability similar to conventional tests, sensitive for cognitive impairment. Should be used as a screening instrument and not as substitute for formal neuropsychological testing
COGDRAS	Wesnes et al, 2010	51	76.5 (6.85)	Mild and moderate AD with acetylcholinesterase inhibitors	Good psychometric properties. Measures of psychomotor speed showed possible sensitivity for detecting decline over 6 months
	Lepeleire, et al., 2005	152	80.01(7.13)	HC	Low sensitivity and specificity of subtests added to PBW battery in diagnosing dementia
Mindstreams (Neurotrax)	Dwlatzky et al., 2010	170	≥60	No impairment (7), questionable impairment (76), very mild (58), mild (26), moderate impairment (2),	Performance differed significantly across groups (p<0.001) poorer overall battery performance for those with greater impairment
	Doninger et al., 2009	27 MCI22HC	MCI: 69.2(6.5)HC: 67.6(4.6)	MCI and HC	MCI performed poorly compared to HC participants in all domains, with significant differences in memory (p= .003; d= 0.96) executive function (p =.046; d=0.64), and overall battery performance (p=.041; d=0.63).
	Fillit et al., 2008	2.888	64.7(18.2)	Neurology (2.539); Primary care (286); Geriatrics (63)	83% rated the test easy to use (p<0.001),.
	Doniger et al.,2006	GDS: 72CSDD: 88	GDS: 74.9 (6.9)CSDD: 78.4 (8.8)	HC, MCI, mild AD	Discriminated among MCI, mild AD, and HC participants following covariation for depression scale score in both cohorts, demonstrates that the battery is unaffected by depression
	Doniger et al.,2005	161	69.1 (9.3)	HC, MCI, mild dementia	Discriminating between demented and non-demented individuals=AUC=0.886 (p<0.001). For discriminating between cognitively healthy individuals and non-cognitively healthy individuals AUC=0.823 (p<0.001)

SD: Standard Deviation; MCI: mild cognitive impairment; aMCI: amnestic mild cognitive impairment; AD: Alzheimer´s disease; FTD: frontotemporal dementia; DLB: dementia with Lewy bodies; HC: healthy control; GDS: Global Depression Scale; CSDD: Cornell Scale for Depression in Dementia.

**Table 2:** Main characteristics of the computerized tests.

Test	Created	Screening for	Domains	Time	Site
CogState	2001	MCI, early Alzheimer’s disease and dementia	Attention, memory, executive function, language, social cognition	15-20 minutes	www.cogstate.com
CNS Vital Signs	2002	MCI	Memory, attention, psychomotor speed, processing speed, cognitive flexibility	30 minutes	https://www.cnsvs.com
COGDRAS		AD and dementias	Attention, concentration, verbal and visuo-spatial recall and recognition, verbal and visuo-spatial working memory, psychomotor speed and information processing speed	30 minutes	
Mindstreams (Neurotrax)	2000	MCI	Memory, executive function, visuospatial, verbal fluency, attention, motor skills, information processing	45-60 minutes	www.neurotrax.com

MCI: Mild Cognitive Impairment; AD: Alzheimer’s disease

**CogState.**^7^ Cogstate Research is a repeatable and sensitive computerized cognitive testing system designed specifically for use in research studies and can evaluate patients aged 6 to 106, which includes: attention deficit hyperactivity disorder (ADHD), fatigue and drug effects, post-operative cognitive dysfunction, MCI, early Alzheimer's disease and dementia, Schizophrenia and mood disorders. CogState was developed as a dementia screening instrument and for assessing concussion and has been shown to be valid, reliable, and sensitive for detecting cognitive impairment.^7,8^ Subtests include measures of simple selection and complex reaction times, continuous monitoring, working memory, matching, incidental learning, and associative learning. The subtests are based on playing card formats and written instructions are presented on screen. Responses are made via a computer keyboard represented graphically on the screen, with responses using the "k" key for yes and "d" key for no. The battery requires 15 to 20 minutes to complete.^1^ Specialized tasks can assess attention, memory, executive function, as well as language and social-emotional cognition if required. In addition to research studies, the batteries of tasks are used in commercial trials to determine the effect of drugs. Minimal learning effects ensure that participants can be tested repeatedly as often as needed, even multiple times in short periods (over a single day).^7,10^

In a study that evaluated healthy older adults (n=105), amnestic MCI (n=48) and AD (n=42) patients over three months, the CogState battery showed high test-retest reliability and stability in all groups and was able to detect AD-related cognitive impairment.^9^

Another study showed that in established dementia, the CogState tasks appeared to be sensitive for detecting cognitive impairment. Repeat administration also provided acceptable stability and test-retest reliability with minimal practice effects at short test-retest intervals even on the same day.^10^ A study comparing dementia (AD, frontotemporal dementia, and dementia with Lewy bodies), MCI, and healthy controls, found the CogState was able to successfully differentiate dementia patients from control subjects, but achieved minimal differentiation between controls and MCI. Repeat administration also provided acceptable stability and test-retest reliability with minimal practice effects at short test-retest intervals.^11^ In a study following healthy older adults over a one-year period, CogState proved to be an instrument that can be used repetitively (at 3, 6, 9 and 12 months), differentiating individuals with risk of increased rates of cognitive decline in memory.^12^

**CNS Vital Signs (VS).**^13^ Developed as a brief clinical evaluation tool, its tests are familiar and well-established: verbal and visual memory, finger tapping, symbol digit coding, Stroop Test, a test of shifting attention and the continuous performance test. Gualtieri and Johnson^13^ published a study of reliability and validity among 1069 individuals aged 7-90 years with psychometric characteristics. The reliability of the tests in the CNSVS battery are very similar to the characteristics of the conventional neuropsychological tests, and the battery was sensitive for detecting the most common causes of cognitive impairment, but should be used as a screening instrument and not as a substitute for formal neuropsychological testing.^13,14^

**Cognitive Drug Research Computerized Assessment System (COGDRAS).**^15^ COGDRAS was developed for use in neuropharmacological research.^15^ A study with 152 older adults that investigated a combination of tests for the diagnosis of dementia using conventional tests and subtests of two computerized battery: the Poon-Baro-Wens (PBW) battery^16^ and COGDRAS, found that computerized tests added very little diagnostic value.^17^

This battery is widely used in clinical trials testing the effect of drugs, mainly on attention.^18^ A study with 51 AD patients assessing the COGDRAS during treatment with acetylcholinesterase inhibitors found this battery has good psychometric properties while the measures of psychomotor speed showed possible sensitivity for detecting decline over 6 months.^19^

**Mindstreams (Neurotrax).**^20^ A computerized testing system for comprehensive clinical assessment of cognitive impairment, designed primarily for use in the elderly. The battery consists of nine subtests: verbal memory, nonverbal memory, Go-No Go response inhibition, Stroop interference, problem solving, visual spatial imagery, verbal rhyming, verbal naming, staged information processing speed, finger tapping, and visuomotor planning.^1^ A study showed the capacity of this battery to discriminate individuals with MCI from cognitively healthy elderly.^20^ Another study by the same author found that the Mindstreams battery provided detailed and distinct cognitive profiles of patients with moderate impairment.^21^

In a study assessing 2888 patients, 83% rated the test as easy-to-use and patients were divided into non–users of computers, patients older than 75, and poor performers.^22^

In a study with a population of 161 older adults divided into healthy, MCI and mild dementia, the Mindstreams was able to discriminate between demented and non-demented individuals=AUC=0.886 (p<0.001) and between cognitively healthy individuals and non-cognitively healthy individuals AUC=0.823 (p<0.001).^22^

In another study, patients were divided into two groups, one assessed by the Global Depression Scale (GDS)^23^ and another by the Cornell Scale for Depression in Dementia (CSDD).^24^ Mindstreams discriminated among MCI, mild AD, and healthy control (HC) participants following covariation for depression scale score in both cohorts, demonstrating that this battery is unaffected by depression.^25^

A study was performed in Afro-Americans, comprising 27 MCI and 22 HC subjects. The MCI patients performed poorly compared to HC participants in all domains, with significant differences in memory (p=.003; d=0.96), executive function (p=.046; d=0.64), and overall battery performance (p=.041; d=0.63).^26^

The Mindstreams battery was used in a study as a gold standard for a validation of the Hebrew version of the Montreal Cognitive Assessment (MoCA Test) as a screening instrument for the early detection of MCI.^27,28^

## DISCUSSION

Some batteries are good for measuring reaction time (CODGRAS) or detecting AD (CogState), while others are for screening (CNS VS) and are easy-to-use and effective for discriminating between healthy controls, MCI and AD (Mindstreams).

Slow reaction time and memory impairment seem to be the main features of cognitive impairment, especially in AD, and clinical markers should be taken into account in the choice of test.^30^

A review article about advances in design for AD in clinical trials compared the most widely used tests, namely, Automated Neuropsychological Assessment Metrics (ANAM),^31^ Computer Assessment of Mild Cognitive Impairment (CAMCI),^32^ CANS-MCI,^33^ CANTAB,^34,35^ CNSVS, Cognitive Drug Research (CDR/COGDRAS), CogState, Cognitive Skills Index (CSI),^36^ MicroCog and Mindstreams (Neurotrax). Strengths and weaknesses were detected for all tests, but the authors were emphatic in affirming that computerized assessment offers several advantages over pen-and-paper tests, in that they have a high degree of standardization in administration and scoring and can measure reaction time accurately.^37^

It is important to highlight the role of the physician or neuropsychologist in cognitive assessment, noting that the computerized test should be considered one more tool to have on hand and not the sole means of reaching the diagnosis.

In conclusion, all CNTs analyzed seemed to be suitable for use in clinical practice. The choice of battery depends on the aspects the clinician wishes to assess, the cost of equipment, and time available. Finally, battery choice also depends on the availability of a version in the language of the subjects studied.

## References

[1] Wild K, Howieson D, Webbe F, Seelye A, Kayen J (2008). Status of computerized cognitive testing in aging: A systematic
review. Alzheimers Dement.

[2] Gualtieri CT, Johson LG (2006). Reability and validity of a computerized neurocognitive test
battery, CNS Vital Signs. Arch Clin Neuropsychol.

[3] Silverberg NB, Ryan LM, Carrillo MC (2011). Assessment of cognition in early dementia. Alzheimers Dement.

[4] Elwood RW (2001). MicroCog: assessment of cognitive functioning. Neuropsychol Rev.

[5] Égerházi A, Berecz R, Bartók E, Degrell I (2007). Automated Neuropsychological Test Battery (CANTAB) in mild
cognitive impairment and in Alzheimer's disease. Prog Neuropsychopharmacol Biol Psychiatry.

[6] Martin TA, Bush S (2005). Ethical challenges with the use of information technology and
telecommunications in neuropsychology. A casebook of ethical challenges in neuropsychology.

[7] CogState The global leader in assessing, monitoring and improving
cognition.

[8] Gualtieri T (2004). Computerized Neurocognitive Testing and its Potential for Modern
Psychiatry. Psychiatry (Edgmont).

[9] Lim YY, Jaeger J, Harrington K (2013). Three-Month Stability of the CogState Brief Battery in Healthy
Older Adults, Mild Cognitive Impairment, and Alzheimer's Disease: Results
from the Australian Imaging, Biomarkers, and Lifestyle-Rate of Change
Substudy (AIBL-ROCS). Arch Clin Neuropsychol.

[10] Hammers D, Spurgeon E, Ryan K (2011). Reliability of Repeated Cognitive Assessment of Dementia Using a
Brief Computerized Battery. Am J Alzheimers Dis Other Demen.

[11] Hammers D, Spurgeon E, Ryan K (2012). Validity of a Brief Computerized Cognitive Screening Test in
Dementia. J Geriatr Psychiatry Neurol.

[12] Darby DG, Pietrzak RH, Fredrickson J (2012). Intraindividual cognitive decline using a brief computerized
cognitive screening test. Alzheimers Dement.

[13] Gualtieri CT, Johnson LG (2005). Neurocognitive testing supports a broader concept of mild
cognitive impairment. Am J Alzheimers Dis Other Demen.

[14] Gualtieri CT, Johnson LG (2006). Reliability and validity of a computerized neurocognitive test
battery, CNS Vital Signs. Arch Clin Neuropsychol.

[15] Simpson PM, Surmon DJ, Wesnes KA, Wilcock GK (1991). The Cognitive Drug Research Computerized Assessment System for
demented patients: a validation study. Int J Geriatr Soc.

[16] Wens L (1993). Impliciet en expliciet geheugen bij dementie van het
Alzheimertype.

[17] De Lepeleire J, Heyrman J, Baro F, Buntinx F (2005). A combination of tests for the diagnosis of dementia had a
significant diagnostic value. J Clin Epidemiol.

[18] Galvin JE, Cornblatt B, Newhouse P (2008). Effects of galantamine on measures of attention: results from 2
clinical trials in Alzheimer disease patients with comparisons to
donepezil. Alzheimer Dis Assoc Disord.

[19] Wesnes K, Edgar C, Andreasen N (2010). Computerized cognition assessment during acetylcholinesterase
inhibitor treatment in Alzheimer's disease. Acta Neurol Scand.

[20] Dwolatzky T, Whitehead V, Doniger GM (2003). Validity of a novel computerized cognitive battery for mild
cognitive impairment. BMC Geriatrics.

[21] Dwolatzky T, Dimant L, Simon ES, Doniger GM (2010). Validity of a short computerized assessment battery for moderate
cognitive impairment and dementia. Int Psychogeriatr.

[22] Fillit HM, Simon ES, Doniger GM, Cummings JL (2008). Practicality of a computerized system for cognitive assessment in
the elderly. Alzheimers Dement.

[23] Yesavage JA, Brink TL, Rose TL (1982). Development and validation of a geriatric depression screening
scale: A preliminary report. J Psychiatr Res.

[24] Alexopoulos GS, Abrams RC, Young RC (1988). Cornell Scale for Depression in Dementia. Biol Psychiatry.

[25] Doniger GM, Dwolatzky T, Zucker DM, Chertkow H, Crystal H, Schweiger A, Simon ES (2006). Computerized cognitive testing battery identifies mild cognitive
impairment and mild dementia even in the presence of depressive
symptoms. Am J Alzheimers Dis Other Demen.

[26] Doniger GM, Zucker DM, Schweiger A (2005). Towards practical cognitive assessment for detection of early
dementia: a 30-minute computerized battery discriminates as well as longer
testing. Curr Alzheimer Res.

[27] Doniger GM, Jo MY, Simon ES, Crystal HA (2009). Computerized cognitive assessment of mild cognitive impairment in
urban African Americans. Am J Alzheimers Dis Other Demen.

[28] Nasreddine ZS, Phillips NA, Bedirian V (2005). The Montreal Cognitive Assessment, MoCA: a brief screening tool
for mild cognitive impairment. J Am Geriatr Soc.

[29] Lifshitz M, Dwolatzky T, Press Y (2012). Validation of the Hebrew version of the MoCA test as a screening
instrument for the early detection of mildcognitive impairment in elderly
individuals. J Geriatr Psychiatry Neurol.

[30] Charchat H, Nitrini R, Caramelli P, Sameshima K (2001). Investigação de marcadores clínicos dos
estágios iniciais da doença de Alzheimer com testes
neuropsicológicos computadorizados. Psicol Reflex Crít.

[31] Levinson D, Reeves D, Watson J, Harrison M (2005). Automated neuropsychological assessment metrics (ANAM) measures
of cognitive effects of Alzheimer's disease. Arch Clin Neuropsychol.

[32] Saxton J, Morrow L, Eschman A, Archer G, Luther J, Zuccolotto A (2009). Computer assessment of mild cognitive impairment. Postgrad Med.

[33] Tornatore JB, Hill E, Laboff J, McGann ME (2005). Selfadministered screening for mild cognitive impairment: Initial
validation of a computerized test battery. J Neuropsychiatr Clin Neurosci.

[34] Fowler KS, Saling MM, Conway EL Semple JM, Louis WJ (1997). Computerized neuropsychological tests in the early detection of
dementia: prospective findings. J Int Neuropsychol Soc.

[35] De Jager CA, Milwain E, Budge M (2002). Early detection of isolated memory deficits in the elderly: the
need for more sensitive neuropsychological tests. Psychol Med.

[36] DeLepeliere J, Heyrman J, Baro F, Buntinx F (2005). A combination of tests for the diagnosis of dementiahad a
significant diagnostic value. J Clin Epidemiol.

[37] Cummings J, Gould H, Zhong K (2012). Advances in designs for Alzheimer's disease clinical
trials. Am J Neurodegener Dis.

